# Combining a Climatic Niche Model of an Invasive Fungus with Its Host Species Distributions to Identify Risks to Natural Assets: *Puccinia psidii* Sensu Lato in Australia

**DOI:** 10.1371/journal.pone.0064479

**Published:** 2013-05-21

**Authors:** Darren J. Kriticos, Louise Morin, Agathe Leriche, Robert C. Anderson, Peter Caley

**Affiliations:** 1 Commonwealth Scientific and Industrial Research Organisation, Ecosystem Sciences, Canberra, Australian Capital Territory, Australia; 2 International Science & Technology Policy & Practice, Department of Applied Economics, University of Minnesota, St. Paul, Minnesota, United States of America; 3 University of Hawai'i, College of Tropical Agriculture and Human Resources, Honolulu, Hawaii, United States of America; 4 Commonwealth Scientific and Industrial Research Organisation, Mathematics Informatics and Statistics, Canberra, Australian Capital Territory, Australia; University of Wisconsin Medical School, United States of America

## Abstract

*Puccinia psidii* sensu lato (s.l.) is an invasive rust fungus threatening a wide range of plant species in the family Myrtaceae. Originating from Central and South America, it has invaded mainland USA and Hawai'i, parts of Asia and Australia. We used CLIMEX to develop a semi-mechanistic global climatic niche model based on new data on the distribution and biology of *P. psidii* s.l. The model was validated using independent distribution data from recently invaded areas in Australia, China and Japan. We combined this model with distribution data of its potential Myrtaceae host plant species present in Australia to identify areas and ecosystems most at risk. Myrtaceaeous species richness, threatened Myrtaceae and eucalypt plantations within the climatically suitable envelope for *P. psidii* s.l in Australia were mapped. Globally the model identifies climatically suitable areas for *P. psidii* s.l. throughout the wet tropics and sub-tropics where moist conditions with moderate temperatures prevail, and also into some cool regions with a mild Mediterranean climate. In Australia, the map of species richness of Myrtaceae within the *P. psidii* s.l. climatic envelope shows areas where epidemics are hypothetically more likely to be frequent and severe. These hotspots for epidemics are along the eastern coast of New South Wales, including the Sydney Basin, in the Brisbane and Cairns areas in Queensland, and in the coastal region from the south of Bunbury to Esperance in Western Australia. This new climatic niche model for *P. psidii* s.l. indicates a higher degree of cold tolerance; and hence a potential range that extends into higher altitudes and latitudes than has been indicated previously. The methods demonstrated here provide some insight into the impacts an invasive species might have within its climatically suited range, and can help inform biosecurity policies regarding the management of its spread and protection of valued threatened assets.

## Introduction

Bioclimatic niche models for invasive alien species (IAS) have become fundamental tools for pest risk assessment (PRA) [1], [2]. They are of most value prior to, or soon after a pest species becomes established in a new region, to delimit the extent of the endangered area within the area of interest of the PRA [3]. This information allows the assets at risk within the PRA area to be identified and quantified, enabling biosecurity managers to decide how best to allocate scarce management resources into pre- and post-border activities to limit the spread of these unwanted organisms, or to initiate eradications. While some attention has been paid recently to methods for quantifying the potential economic impact IAS might have on productive assets such as plantation forests [4], we are unaware of any efforts to identify systematically assets of particular biological conservation concern within areas threatened by IAS.

*Puccinia psidii* sensu lato (s.l.) is a plant pathogenic rust fungus native to South and Central America, and possibly the Caribbean, that is commonly known as guava or eucalyptus rust [5], [6]. It was first recorded outside its native range in Florida, USA in the late 1970s [7]. More recently it was discovered in 2005 in California [8] and Hawai'i, USA [9], Japan in 2007 [10], China in 2009 [11] and Australia in 2010 [12], where it was originally identified as *Uredo rangelii* (commonly referred to as myrtle rust) [13]. *Puccinia psidii* s.l. is most feared because it has a very wide host range within the family Myrtaceae [14]. A recent study with an accession of *P. psidii* s.l. from Australia has shown that it can infect species across all 15 tribes of the subfamily Myrtoideae in the family Myrtaceae present in Australia [14]. Based on pathogenicity tests, there is evidence that several ‘races’ (‘strains’) exist within *P. psidii* s.l., each with a slightly different host range [14], [15], [16], [17], but a comprehensive inventory of the total number of races has never been undertaken. Possible differences in climatic preference between these races have not been investigated. The rust only infects young, actively growing foliage of plants, and as a result has more adverse impacts on young plants [6]. Severe damage has been reported in the field in some years on a range of hosts in both the native and introduced ranges [6], [15]. Considering the dominance of myrtaceaeous species in Australia, there is great concern that recurrent epidemics of *P. psidii* s.l. could transform many of the major natural ecosystems and forestry plantations.

There have been at least three previous published attempts to estimate the geographic invasive potential of *P. psidii* s.l. Booth et al. [18] used a homoclime analysis, with a special focus on Australia. This model was subsequently revised and results and methods published in Glen et al. [6] and Booth and Jovanovic [19], respectively. Magarey et al. [20] used NAPPFAST (NCSU APHIS Plant Pest Forecasting System), to estimate the global potential for *P. psidii* s.l. infection based on its known distribution in South America and the Caribbean. Most recently, Elith et al. [21] used the MaxEnt model to explore the effects of taxonomic uncertainty on the potential range of *P. psidii* s.l. using known distribution data, including some from Hawai'i. While these models provided initial indications of the potential distribution of the rust, they were either based on empirical biological knowledge of the organism's response to environmental variables [20], or its known distribution [18], [21], but not both, and therefore may be sub-optimal.

There are few methods that are well-equipped for estimating the potential distribution of an organism in a novel environment such as on a new continent or under climate change scenarios [22], [23]. CLIMEX [24], [25] is one of these methods. It has become a popular climatic niche modelling tool because it is very well suited to modelling the potential distribution of invasive organisms, allowing the modeller to take advantage of knowledge of the biology and phenology of the organism, as well as its geographical distribution. By considering information from various knowledge domains, it is possible to robustly cross-validate parameter estimates, increasing confidence in the resulting models. CLIMEX Compare Locations models have been used successfully to estimate the potential distribution and relative climate suitability of a range of plant pathogens (e.g., [26], [27], [28], [29], [30], [31]).

The recent invasion of California, Hawai'i, Japan, China and Australia [8], [9], [10], [11], [12], and the earlier invasion of Florida [7] by *P. psidii* s.l. present opportunities to study in greater detail the geographic and climatic limitations for the establishment of this pathogen. Distribution data from Hawai'i are particularly useful because the extremely steep climatic gradients cover a wide range of conditions within a very limited area. Further, Hawai'i also has some very susceptible hosts to *P. psidii* s.l. that are widespread. Despite being present in Hawai'i for a relatively short period of time, it is likely that *P. psidii* s.l. has had the opportunity to spread to all suitable climates available within the area. Given that the known distribution of *P. psidii* s.l. is primarily tropical, the highest altitude location records on the islands of Hawai'i are of most interest to biosecurity agencies, as these could indicate the cold tolerance limits for the pathogen. A caution in making use of this information is that Hawai'i has relatively few climate stations sampling very steep climatic gradients. The temperature variables are the most critical here. Fortunately, these are strongly influenced by altitude, so it is possible to use splining techniques to interpolate climatic averages reasonably accurately [32].

In this study, we firstly gathered data on the worldwide distribution of *P. psidii* s.l., including its distribution in regions where recent incursions have occurred – California (2005), Hawai'i (2005), Japan (2007), China (2009) and Australia (2010) [8], [9], [10], [11], [12]. A laboratory experiment to gather biological data on an accession of *P. psidii* s.l. from Australia was also performed to add to existing published data on other accessions from Brazil [33], [34]. The distribution data, except for Australia, China and Japan, was then combined with all empirical biological information available into the CLIMEX modelling software to develop an improved climatic niche model and generate more robust estimates of the rust's potential distribution worldwide. The fit of the model was tested using the Australian distribution data and the point records in China and Japan. Finally, we used Australia as a case study to demonstrate the benefits of combining the CLIMEX model of *P. psidii* s.l. with the distribution of potential Myrtaceae host plants, including threatened species and forestry plantations, to identify areas that are most at risk from the rust.

## Methods

### Effect of temperature on *Puccinia psidii* s.l. urediniospore germination on agar disks

Urediniospores of a single-uredium isolate of *P. psidii* s.l. (DAR 81284) were produced on *Syzygium jambos* plants, and harvested as described in Morin et al. [14]. A small amount of spores was mixed with liquid paraffin oil (previously found to have stimulatory effects on germination; [35]) and the suspension was applied with a fine camel hair paint brush onto the surface of 8 mm diameter disks of 2% water agar (Merck) with the aid of a dissecting microscope. The water agar provided the necessary moisture required for spore germination [36]. The spore suspension density was adjusted prior to application by adding more oil to ensure that spores were well separated from each other on the agar once applied. Thirty-two inoculated disks were placed in each of the base of seven 90 mm diameter plastic Petri dishes. A four by eight grid pattern with cells numbered from 1 to 32 printed on paper was fixed on the outside of each dish base to provide a unique identification number to each inoculated disk. Each Petri dish was then wrapped in foil and placed in one of seven different compartments of a uni-directional temperature gradient plate similar to that of Barbour and Racine [37]. The Perspex compartments experienced the following average temperatures (standard deviation): 8.8°C (0.19), 11.2°C (0.20), 15.6°C (0.25), 19.1°C (0.35), 21.8°C (0.32), 26.9°C (0.38) and 29.7°C (0.74). The recorded temperature in each compartment was based on measurements taken over a three week period with a Hobo data logger (Onset Computer Corporation). Four disks (replicates) per plate were removed at random (based on randomly generated numbers) at 3, 6, 9, 12, 18, 24, 30 and 42 h after the commencement of the experiment and each placed on a drop of lacto-glycerol blue stain on a glass slide. The lacto-glycerol blue was absorbed rapidly by the disks, and germination was arrested without disturbance of the disk upper surface. Slides with stained disks were stored in large Petri dishes lined with moist paper towel and placed in a refrigerator until microscopic assessment was completed. For each disk several non-selectively chosen fields of view were examined using a compound microscope until the germination of a total of 100 urediniospores had been assessed. Urediniospores were considered to have germinated when the length of the germ-tube was greater than half the width of the spore. The experiment was performed twice.

Data analysis was performed using the statistical package R (release 2.13.0) [38]. Germination proportion data were analysed using a generalised additive mixed-effects (GAM) model [39], [40], assuming a binomial error with a log link function to estimate response curves with estimated temperature and duration on agar disks as covariates. Trial number was included in the model as a random effect. The model included interactions between temperature and duration on agar disks, allowing for two-dimensional smoothing. A scale-invariant tensor product smooth, which uses a lattice of bendy strips with different flexibility in different directions was employed [39]. This smoother was chosen as temperature and duration on agar disks are anistropic (measured in different units), hence it is appropriate for the degree of smoothing to differ between them (c.f. isotropic smoothers).

### Geographical distribution of *Puccinia psidii* s.l

**Figure 1:**
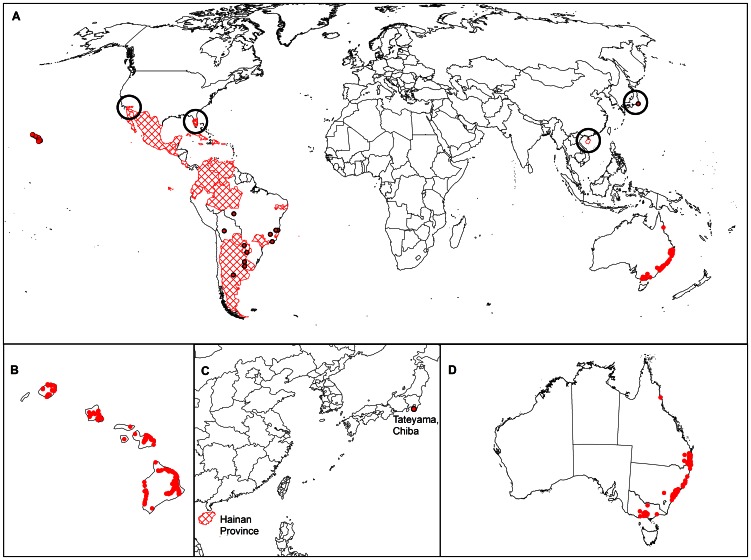
Global distribution of *Puccinia psidii* s.l. based on most recent literature and records from Australia, as of April 2012 (A). Call-outs of distribution in Hawai'i (B), Asia (C) and Australia (D). Dots indicate point location records. Cross-hatched areas indicate administrative regions. Black circles draw attention to small administrative regions where the fungus has been recorded.

The historical distribution of *P. psidii* s.l. used to build the CLIMEX model was assembled from several sources. Booth et al. [18] reports nine point locations where the rust has been recorded in South America. Unpublished data on counties or locations where the rust has been recorded in Florida, California and Hawai'i were obtained from a range of collaborators (personal communications: Anne Marie La Rosa, United States Forest Service and Cherisa Coles, Janice Uchida and Mee Sook Kim, University of Hawai'i). Additional data on the distribution in Hawai'i were obtained from a roadside survey on the islands of Kaua'i, O'ahu, Maui and Hawai'i [41]. The country-level occurrence records of *P. psidii* s.l. comprised in the CABI Distribution Maps of Plant Diseases [42] and the point distribution records indicated in Magarey et al. [20] represent the centroids of countries or regions, and not true point locations, limiting their value for model calibration. The single point records in Japan and China, reported by Kawanishi et al. [10] and Zhuang and Wei [11], respectively, and the distribution records for Australia, as of April 2012, that had been collected separately by the Queensland, New South Wales (NSW) and Victorian State Governments and kindly provided by various agencies, were not used to build the model but rather used to validate the model fit. The known global distribution of *P. psidii* s.l. based on all these sources is presented graphically in Figure 1.

### Base climate data and CLIMEX model

The primary base climatology used to build the model was the CliMond 10′ climate normals centred on 1975 (CM10_1975H_V1_1) [43]. We chose to use this hybrid dataset as it provided improved spatial resolution over the traditional 0.5 degree climate datasets, and improved data quality. We attempted to supplement the available data for Hawai'i through the US National Climatic Data Centre and the United States Geological Survey, because the existing climate data is very scant, and on these islands the climatic gradients are extremely steep for both rainfall and temperature. While it was possible to supplement the data with additional temperature, and to a lesser extent rainfall records, it was impossible to locate any additional source of data variables to indicate atmospheric wetness (e.g., absolute humidity, relative humidity, vapour pressure, or vapour pressure deficit). In an attempt to overcome this problem, a new set of surfaces were generated using NewLocClim [44]. A digital elevation model (DEM) was developed from the Shuttle Radar Topography Mission data [45] by extracting values for a 0.025 degree regular grid of points within the land areas of the State of Hawai'i. This DEM was provided to NewLocClim as a means of adjusting the interpolation of meteorological station data to account for topographic variation. The thin-plate spline was selected as the interpolation method as this has been used successfully in Australia and elsewhere to produce high quality climate surfaces [46], [47]. The resulting surfaces for Hawai'i appear to represent the temperature surfaces and the rainfall for the wetter areas adequately; however, the representation of precipitation in the drier areas is not satisfactory when compared with the Rainfall Atlas of Hawai'i [48].

The CLIMEX niche modelling package [24], [25] was used to create a model for *P. psidii* s.l. (Table 1). The model is based on previous work [49], in which the model stresses were fitted before location records in Japan, China and Australia had been collected. In the model presented in this paper, only the growth index parameters were adjusted to accommodate the observed germination responses of urediniospores of an Australian accession of *P. psidii* s.l. to a range of temperatures under experimental conditions.

**Table 1:** CLIMEX Compare Locations model parameters for *Puccinia psidii* s.l. (mnemonics are taken from Sutherst et al. [25]).

Index	Parameter	Value^a^
Temperature	DV0 = lower threshold	10°C
	DV1 = lower optimum temperature	14°C
	DV2 = upper optimum temperature	25°C
	DV3 = upper threshold	32°C
Moisture	SM0 = lower soil moisture threshold	0.24
	SM1 = lower optimum soil moisture	1
	SM2 = upper optimum soil moisture	1.5
	SM3 = upper soil moisture threshold	2
Cold stress	TTCS = temperature threshold	2.5°C
	THCS = stress accumulation rate	−0.0045 Week^−1^
	DTCS = degree day threshold	17°C days
	DHCS = stress accumulation rate	−0.0015 week^−1^
Hot stress	TTHS = temperature threshold	32°C
	THHS = stress accumulation rate	0.002 Week^−1^
Dry stress	SMDS = soil moisture threshold	0.2
	HDS = stress accumulation rate	−0.012 Week^−1^
Annual Heat Sum	PDD = degree-day threshold^b^	980°C Days

a Values without units are a dimensionless index of a 100 mm single bucket soil moisture profile.

b Minimum annual total number of degree-days above DV0 needed for population persistence.

The Compare Locations function in CLIMEX calculates an annual index of climatic suitability, the Ecoclimatic Index (EI), which reflects the combined potential for population growth during favourable periods and survival during stressful periods (Equation 1). The annual Growth Index (GI_A_) describes the potential for growth of the modelled organism as a function of average weekly soil moisture (Moisture Index; MI) and temperature (Temperature Index; TI) during favourable conditions only (Equation 2; GI_W_ = TI_W_×MI_W_). Stress indices describing cold (CS), wet (WS), hot (HS), and dry (DS) and their interactions with one another can be used to describe the population response to climatically unfavourable conditions. The individual components of stress are combined into a stress index (SI) and a stress interaction index (SX) (Equations 3 and 4; CDX = Cold-Dry Stress, CWX = Cold-Wet Stress, HDX = Hot-Dry Stress and HWX = Hot-Wet Stress) [25].
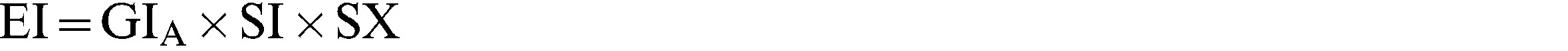
(1)

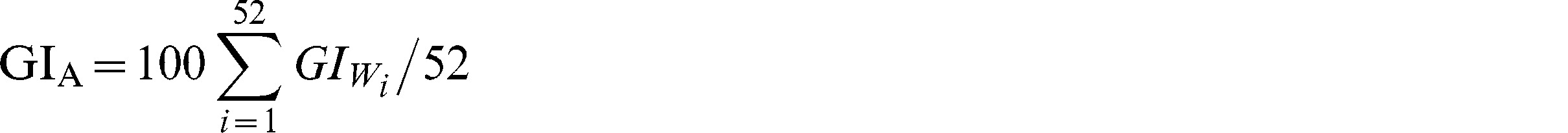
(2)

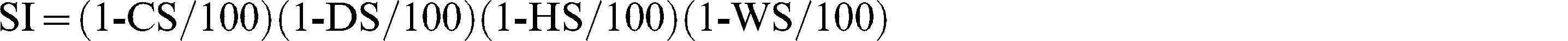
(3)

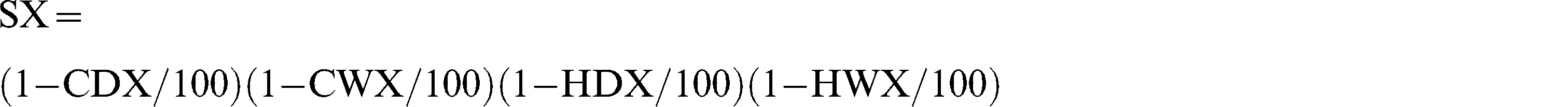
(4)


The three main sources of information for fitting the Temperature Index function were the ecophysiological observations of Ruiz et al. [33] and Ferreira [34] and those obtained in the laboratory experiment with the Australian accession of *P. psidii* s.l. included in this paper. Values for the Temperature Index parameters suggested by the experiment reported here are somewhat lower than the values indicated by experiments using Brazilian accessions of the rust [33], [34]. The parameters adopted in the model span results from all three sets of experiments, taking the lower values from the Australian accession, and the upper values from the Brazilian accessions. The resulting climate suitability maps therefore portray the combined risks from all known sources. Using these parameters it is apparent that the low temperature requirements for germination are not limiting the distribution of *P. psidii* s.l. because the fitted cold stress temperature threshold (TTCS) is considerably different to the experimentally-estimated minimum temperature for development (DV0).

While the Growth Index components are best informed by direct experimental observations [50] or inferred from phenological observations [51], stresses, which indicate negative population growth, are best inferred from distribution data; though critical thresholds can still be informed by experiments and phenological observations. The stress parameters were adjusted until the EI value was positive at all recorded locations. As explained above, the country level data reported in the CABI Distribution Maps of Plant Diseases [42] and elsewhere [20] were not used directly in the model fitting as they were too coarse to guide parameter selection. The role of these records in the modelling process is to provide some level of “fuzzy logic” model verification: where model and data agreement required that somewhere in each of the countries that have reliable presence records is projected to be climatically suitable.

The soil moisture indices were set in consideration of the distribution of the rust in Hawai'i and Argentina. The upper soil moisture values were adjusted to allow *P. psidii* s.l. to thrive in the extremely wet areas to the north of Hilo on the island of Hawai'i, where the rust has been recorded. The lower soil moisture threshold for development (SM0) was adjusted downwards to allow it to persist barely at Misiones in south western Argentina, at the dry end of its distribution (Fig. 1).

The minimum annual heat sum for population reproduction (980 degree days above DV0) was set to allow persistence at the highest elevation location record on the island of Maui in Hawai'i. Two forms of cold stress were employed in this model to limit further its potential to persist in cold climates. A monthly average daily minimum of 2.5°C is associated with frost events (about one per week). This limit for TTCS is probably associated with the temperature tolerances of the known hosts, rather than a direct physiological impact on *P. psidii* s.l. A degree day cold stress (DTCS) was also employed in order to achieve a satisfactory fit to the known distribution data. The DTCS threshold of 17 degree days per week above DV0 (10°C) suggests that the pathosystem needs to be actively growing by a small amount each day in order to offset respiration losses; extended periods with insufficient heat could lead to population reductions as energy reserves are run down. Cold stress was used to limit the potential range of *P. psidii* s.l. near the single Bolivian record (Fig. 1). Using these inferred parameters, the model also limits the range about 100 km south west of the westernmost location record in Argentina (Misiones). The spatial independence of these extreme range records provides some additional confidence in this limit.

Dry stress is likely to affect the pathogen indirectly, through the plant host; reducing turgor and the availability of photosynthate, and ultimately, the availability of plant hosts. We infer dry stress by virtue of the fact that *P. psidii* s.l. has not been recorded in areas experiencing drought stress, and from the general observation that it requires the presence of plant hosts that are sensitive to drought stress. Accordingly, the dry stress parameters were set to allow marginally suitable persistence at the driest location record on the island of O'ahu, Hawai'i. Using these parameters, there is a considerable amount of dry stress (64%) at the single high elevation location record in Bolivia (Fig. 1). This is consistent with ecophysiological expectations; given the credibility of the record, and the dry winters in this location, modelled dry stress should be present, but non-lethal at this point in Bolivia.

On the island of Hawai'i, *P. psidii* s.l. is present in all areas sampled throughout the area west of Hilo on the eastern side of the island (Fig. 1B), which, according to Giambelluca et al. [48] receives extremely large rainfall totals throughout the year. Accordingly, wet stress parameters were not used to limit the range of *P. psidii* s.l. The effect of hot-wet stress as a limit on the range extent in the Amazon basin was investigated. Using hot-wet stress to limit the distribution to the western most point location in Brazil had the undesirable effect of making Florida appear unsuitable. It also severely limited the potential range in Amazonas province in Brazil, where the rust has been recorded, albeit imprecisely (Fig. 1) [42]. In view of these results, it was decided to remove hot-wet stress from the model, accepting that this aspect must be treated with caution as it is possible that the model overestimates the potential for growth under very warm and wet conditions.

The CLIMEX model was partially validated using records from Japan, China and Australia, by assessing the model sensitivity numerically. Due to the unstable nature of the range of an invasive species, it is not possible to satisfy the data requirements for a formal assessment of model specificity (i.e. the proportion of false positives), hence the model specificity was only assessed subjectively.

### Distribution of potential Myrtaceae hosts within the modelled climatic envelope in Australia

#### Geographical patterns of species richness

The geographical distribution of potential Myrtaceae hosts of *P. psidii* s.l. in Australia was gauged by querying the Australian Virtual Herbarium [52], and selecting all 197 491 records where Family equalled ‘Myrtaceae’, and the latitude and longitude coordinates fell within the area where *P. psidii* s.l. was modelled as having an EI>0. To estimate the geographical patterns of species richness of the putative host plants of *P. psidii* s.l. at the scale used for the climate modelling, the number of species records within each cell was summed, ignoring multiple records for the same species within each cell. Because of the fluid and inconsistent nature of the taxonomic classification of records at the sub-specific level, only genus and species epithets were considered when determining taxonomic uniqueness within each cell. To avoid biasing the diversity counts within each cell, where the species epithet was blank (1 926 records) or ‘sp’ (940 records) in the extracted herbarium dataset, the species field was filled with ‘sp.’ using the search and replace option in Microsoft Excel. The Myrtaceae species location records were then imported into ArcGIS 10.3 (ESRI, Redlands, Ca., USA) and spatially joined to the cell identifiers for the corresponding 10′ climate data, before being re-exported to Microsoft Excel, where the Remove Duplicates option was employed. A total of 121 377 non-unique combinations of genus, species and cell identifier were found and removed, leaving 59 814 taxonomically and geographically unique records. The unique records were re-imported into ArcGIS and spatially joined to the climate dataset. The species richness for Myrtaceae in each 10′ climate cell was then gauged by dividing the number of taxonomically unique records in each 10′ climate grid cell by the projected area in km^2^.

#### Threatened Myrtaceae species

A map of the Myrtaceae species listed as threatened under the Australian Government Environment Protection and Biodiversity Conservation (EPBC) Act [53] that fall within the area where *P. psidii* s.l. was modelled as having an EI>0 was generated as above.

#### Hardwood plantations

The major hardwood (Myrtaceae) and mixed hardwood and softwood forest plantations present in Australia were extracted from the Australian National Forest Inventory for 2010 [54]. The dataset was projected using an Albers equal area projection to estimate areas for the forest plantations. These data were spatially intersected with the CLIMEX model results and the Australian State boundaries to estimate the areas of hardwood and mixed hardwood and softwood plantations climatically suitable for supporting the establishment of populations of *P. psidii* s.l. based on the EI. For mapping purposes, the 10′ grid cells were used to indicate the presence of any hardwood forest coups identified in the Australian Land Use dataset V4 [55].

## Results

### Effect of temperature on *Puccinia psidii* s.l. urediniospore germination on agar disks

**Figure 2:**
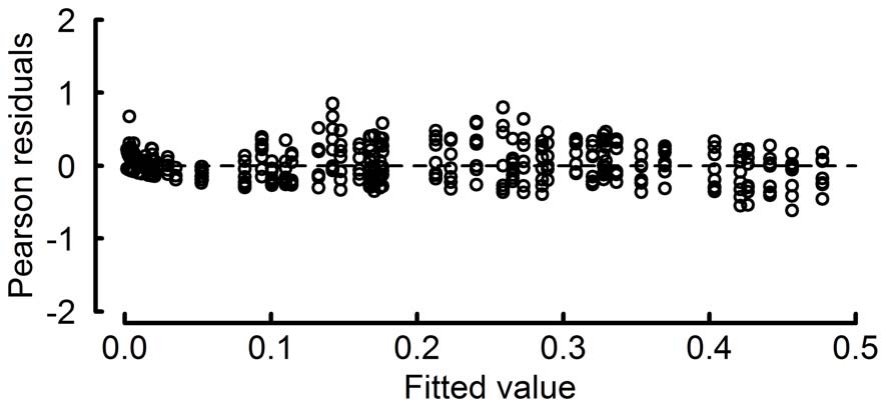
Pearson residuals (the difference between the observed and fitted proportions divided by the standard error of the fitted proportion) versus fitted model values for the GAM model of the effect of temperature and duration on agar disks on germination of *Puccinia psidii* s.l. urediniospores.

**Figure 3:**
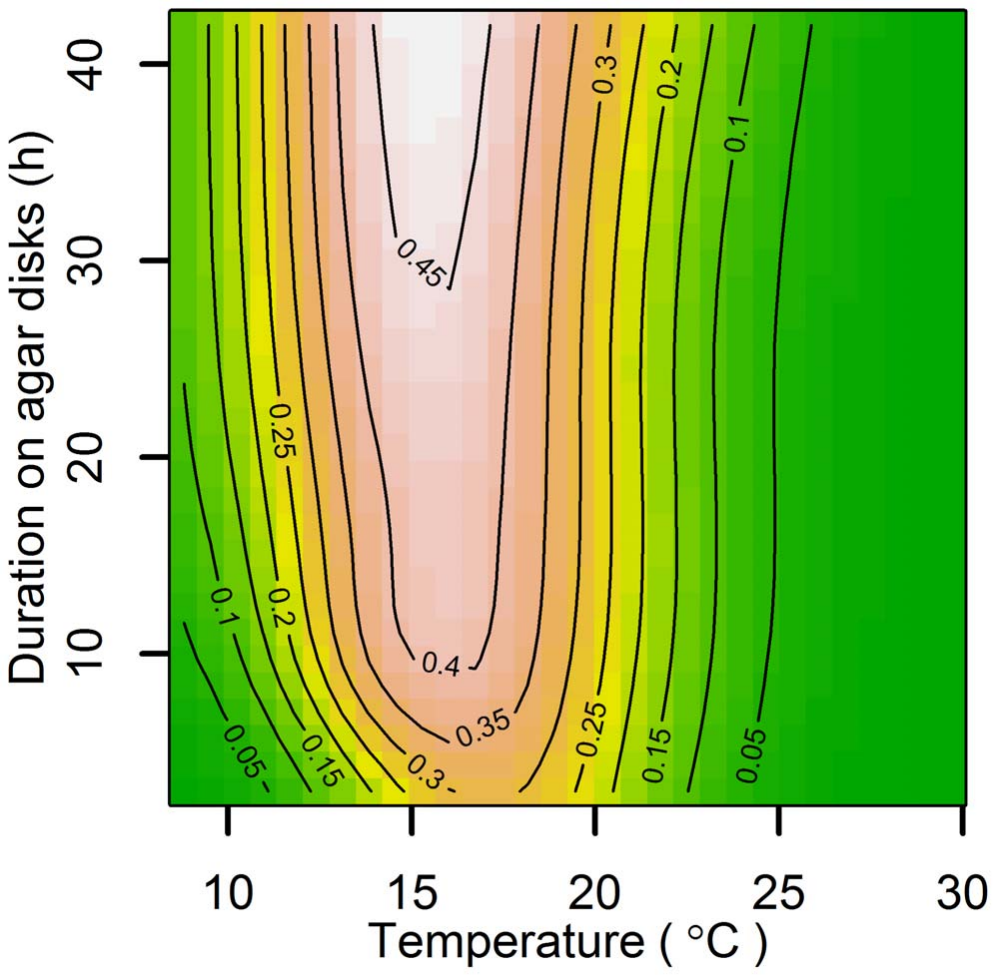
Effect of temperature on germination of urediniospores of *Puccinia psidii* s.l. (Australian accession; DAR 81284) on agar disks, which provided the necessary moisture for germination. Proportion of spores that germinated at each treatment combination (seven temperatures × eight durations on agar disks, and four replicates per treatment combination in each trial) is represented by the isolines (proportion of germinated urediniospores increases from dark green to pale orange). Results are pooled from two trials.

The smooth terms (temperature and duration on agar disks, which correlates with exposure to moisture required for germination) in the model were highly significant based on approximate measures of significance (F_18,18_ = 85.2, P<0.001). More importantly, a plot of Pearson residuals (the difference between the observed and fitted proportions divided by the standard error of the fitted proportion) versus fitted model values revealed no bias or outliers (Fig. 2); meaning that the GAM model fitted the observed germination data very well over a wide range of germination probabilities. Germination percentages were overall higher in the first trial compared to the second trial, but the response trend to treatments was similar for the two trials. At most temperatures, urediniospores began to germinate within 3 h of application onto the agar surface, although a preferred temperature range was already evident (Fig. 3). After 6 h on agar disks, germination had already reached 35% at temperatures between approximately 15 and 18°C. It continued to increase slightly as the period on agar disks extended until it reached a maximum of 45% germination after more than 30 h on agar disks. Germination occurred over the complete range of temperatures tested (8.8–29.7°C), but was the highest (>35%) between temperatures of approximately 12 and 20°C (Fig. 3). The lower temperature threshold for germination observed here is considerably lower than that indicated by the experiments of Ruiz et al. [33] and Ferreira [34]. Accordingly, DV0 was adjusted downwards to 10°C, leaving allowance for the moderating effect of the averaging process in calculating the long-term climate data compared with the instantaneous measurements in the laboratory studies.

### CLIMEX model

**Figure 4:**
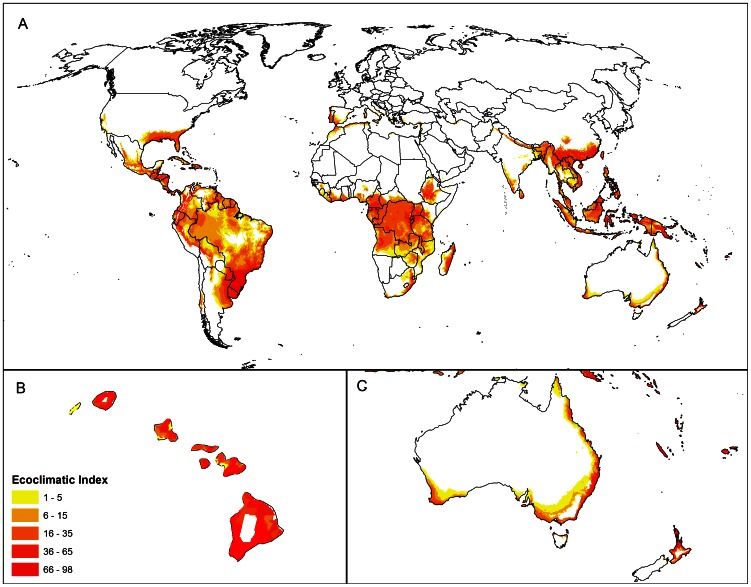
Relative climate suitability for *Puccinia psidii* s.l. as indicated by the CLIMEX Ecoclimatic Index (EI) modelled using the CliMond CM10 1975H V1_1 climate dataset [43]. A) World, B) Hawai'i, and C) Australasia. The EI reflects the combined potential for population growth during favourable periods and survival during stressful periods. Values of zero indicate no potential for establishment, while values of 100 indicate optimal conditions for growth and survival year round.

The CLIMEX EI world map of *P. psidii* s.l. shows its preference for moist climates with moderate temperatures throughout the wet tropics and sub-tropics, and even extending into some cool regions with a mild Mediterranean climate (Fig. 4a).

In the native range, the regions of highest climatic suitability fall in Brazil and Paraguay in south-eastern South America, and also in a narrow band in the north-west of South America. The Caribbean and surrounding moist regions of Central America and the invaded areas of the USA are also highly climatically suitable. Its range appears limited by cold and dry stress in the south and western parts of Argentina, dry stress near the Rio Grande River in New Mexico and Texas in the USA, and by cold stress elsewhere in the USA (data not shown).

In other regions where *P. psidii* s.l. has invaded, there are several, highly climatically favourable areas throughout the State of Hawai'i (except for the saddle on the main island of Hawai'i, Fig. 4b) and most of South-East Asia (Fig. 4a). In Eastern and Southern Asia, it is only limited by cold stress in northern China, and hot and dry stress in India, respectively. In Australia (Fig. 4c), favourable climates are restricted mostly to near-coastal southern and eastern regions where it is limited by cold stress (southern parts of the Great Diving Range), and by hot and dry stress in the interior (data not shown).

Suitable climates for *P. psidii* s.l. can also be found in some of the regions that have not yet been invaded by the rust. Highly favourable climates exist in coastal parts of the Mediterranean (restricted by hot and dry stress), high elevation areas throughout Central Africa, and eastern coastal regions of South Africa and Madagascar (Fig. 4a). In Australasia, the island chains of the Solomon Islands, New Caledonia and Vanuatu are also highly suitable climatically (Fig. 4c). In New Zealand, much of the North Island and a very small area in the north of the South Island are climatically suitable.

**Figure 5:**
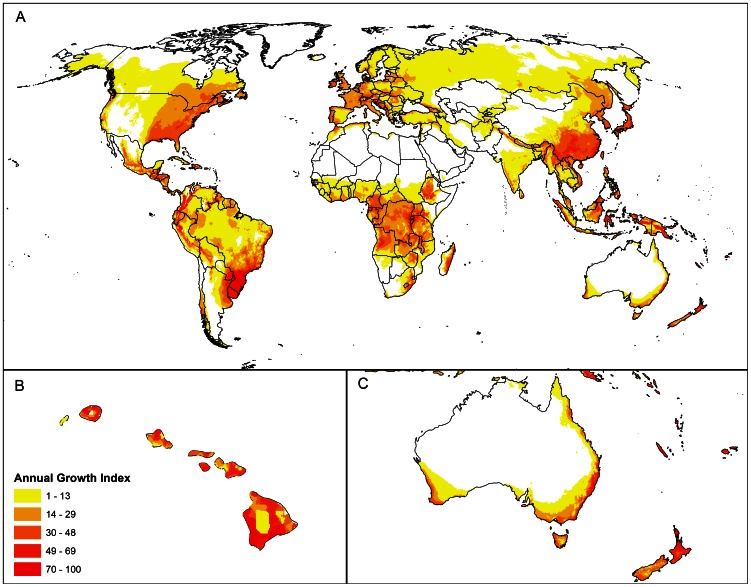
Relative growth potential for *Puccinia psidii* s.l. as indicated by the CLIMEX annual Growth Index (GI_A_) modelled using the CliMond CM10 1975H V1_1 climate dataset [43]. A) World, B) Hawai'i, and C) Australasia. The GI_A_ summarises the potential for population growth across the year, ignoring the effects of stresses and minimum annual heat sum requirements to complete a generation. Outside of the area of suitable climate indicated by the Ecoclimatic Index (Fig. 4), the GI_A_ estimates the relative potential for infection and population growth.

The potential for *P. psidii* s.l. to infect and grow under favourable conditions only, without taking into account potential survival during stressful periods, is indicated by the GI_A_ (Fig. 5). A positive GI_A_ occurs in many areas depicted in the CLIMEX EI map (Fig. 4) as being unsuitable for persistence. This situation indicates that the amount of population growth during the favourable season(s) is insufficient to offset the population declines during the stressful season(s). The ongoing presence of *P. psidii* s.l. in these areas would depend on seasonal reinvasion from nearby source areas.

### Model Validation

The CLIMEX EI world map shows that all known recorded point locations for *P. psidii* s.l. are modelled as being climatically suitable, and all region records include at least some locations that are modelled as being climatically suitable (Figs 1, 4a), indicating perfect model sensitivity (i.e. sensitivity = 1). This includes all records in Australia, China and Japan reserved from model fitting, providing strong validation of the model. The cold and dry stress constraints appear to be well supported with independent point locations in North and South America and Hawai'i lying on similar cool limits. The model specificity is satisfactory. The areas indicated as climatically suitable, but where there are no records (e.g., central Africa), appear ecologically plausible, sharing a similar climate to areas that are presently occupied by *P. psidii* s.l. During the initial model-fitting, there was evidence that *P. psidii* s.l. was found in slightly cooler locations in its introduced range (e.g., in southern California), than was known from within its presumed native range in South America and the Caribbean. However, in the absence of more extensive surveys such as gradsect trapping [56], the potential exists for *P. psidii* s.l. to be present, but undetected in cooler locations in the native range.

### Distribution of potential Myrtaceae hosts within the modelled climatic envelop in Australia

**Figure 6:**
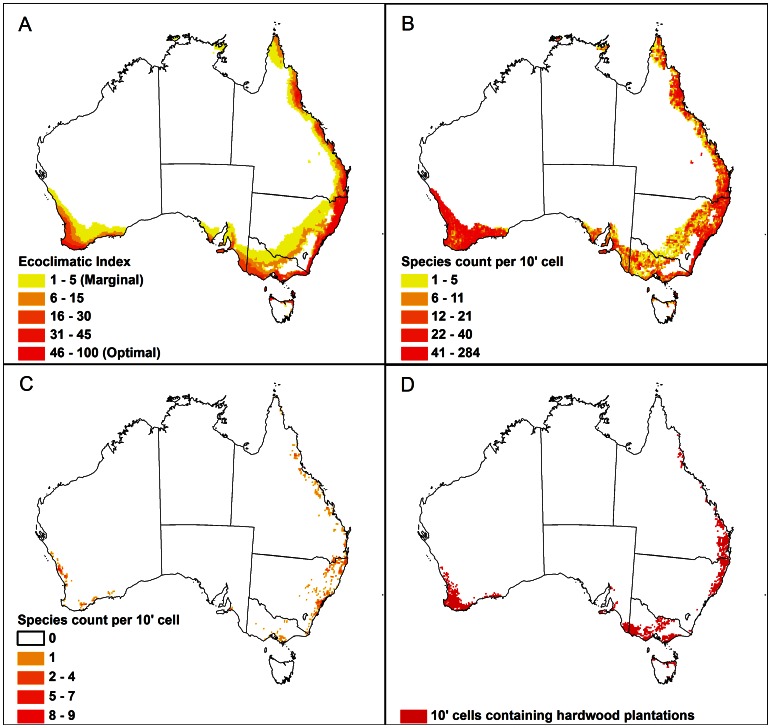
Australian natural assets at risk from *Puccina psidii* s.l. Comparison of the climate suitability map for *Puccinia psidii* s.l. in Australia as indicated by the CLIMEX Ecoclimatic Index (A) with the geographical distribution of potential Myrtaceae hosts within the climate suitability envelop. B) Species richness of Myrtaceae (species per 10′ cell), C) density of threatened species {unique records of species listed under the Environmental Protection and Biodiversity Conservation Act 1999 (Australia) [53]}, and D) 10′ cells containing hardwood and mixed hardwood and softwood forest plantations.

In Australia, pockets of high densities of myrtaceous species (Fig. 6b) are scattered throughout the area climatically suitable for persistence of *P. psidii* s.l. (EI>0) (Figs 4c or 6a). The highest species richness of myrtaceous hosts is found in four main areas: in descending order, the south west of Western Australia (WA), the coastal wet tropics and south-eastern region of Queensland and the coastal hinterland of central and northern NSW (Fig. 6b). Within the modelled climatic envelop, the greatest density of threatened Myrtaceae species listed under the EPBC Act falls in 1) the Sydney Basin and the eastern border region of NSW and Queensland, 2) a small western coastal region to the south of Geraldton in WA, and 3) a small south coastal region of WA between Albany and Esperance (Fig. 6c).

Most of the hardwood forestry operations in Australia fall within the climatic potential range for *P. psidii* s.l. to persist (EI>0) (Table 2). The greatest concentrations of forests at risk are in southern and south-western parts of WA, south-eastern South Australia, throughout the mesic midlands of Victoria and coastal northern NSW and south-eastern Queensland (Fig. 6d, Table 2). Small pockets also exist in the northern extremes of Tasmania and the wet tropics of northern Queensland (Fig. 6d).

**Table 2:** Areas of hardwood and mixed hardwood and softwood plantations in Australia by State and Territory that fall within the climatic potential range for *Puccinia psidii* s.l. (Ecoclimatic Index>0) (Areas calculated from Australia's plantations inventory data [54]).

State	Area at risk of establishment (ha)	Area where establishment is unlikely (ha)	Total plantation area (ha)^b^	Proportion of total plantation area at risk (%)
Northern Territory	0	762	762	0
Tasmania	40 607	191 648	232 254	17.5
New South Wales	83 453	2 172	85 625	97.5
Queensland	26 569	469	27 038	98.3
South Australia	58 385	4	58 393	100.0
Victoria	187 781	19 478	207 259	90.6
Western Australia	289 295	1 435	290 730	99.5
Total	686 090	215 972	902 062	76.1

NB. Total areas do not equal those quoted in [54] due to the exclusion in this analysis of data supplied to the National Forest Inventory in tabular form at the State or Territory level. For the same reason, data are not available for the Australian Capital Territory.

## Discussion

### Potential Host Risks

The family Myrtaceae comprises a large number of species that are widespread across the Australian continent, often occurring as major components of natural ecosystems [57], [58]. *Eucalyptus* spp. are among the most widely planted species in the family Myrtaceae and are popular in many parts of the world as forestry resources with more than 20 million ha planted (e.g., Australia, Brazil, China, India, South Africa and Thailand) [59]. They are also valuable water-use-efficient amenity trees grown in many areas of the world (e.g., California) [60]. In some places eucalypts have even taken on an iconic character (e.g., on the Argentine pampas they have frequently been planted along roadsides) [61]. Based on our model, these regions all appear climatically suited to *P. psidii* s.l., and depending on host suitability, the eucalyptus assets of regions that have not yet been invaded by the rust may be at risk if dispersal barriers are overcome.

Given the rapid spread of *P. psidii* s.l. to Japan, China and Australia in the last few years, and the large spatial gaps involved, it would seem inevitable that the identified uninvaded risk areas could become invaded eventually. Biosecurity efforts should therefore be aimed at slowing its global spread with, for example, the use of relatively low-cost phytosanitary precautions, and the development and deployment of resistant host varieties, rather than attempting to prevent its spread through quarantine barriers. In regions where Myrtaceae are non-native, the numbers of valuable myrtaceaeous species are limited. This makes it potentially feasible to undertake programmes to develop and deploy plant varieties that are resistant to *P. psidii* s.l. In Brazil, for example, before resistant clones were deployed, *P. psidii* s.l. was reported to cause major damage in eucalyptus plantations, particularly in the first two years of planting [62]. The existence of different pathotypes of *P. psidii* s.l. (e.g. [15], [16], [17]) however, means that any attempt to manage the invasion risks using resistant hosts should, if possible, confirm that resistance is effective across the known pathotypes.

In Australia, the key concerns associated with the recent invasion by *P. psidii* s.l. include the additional risks to native species and ecosystems important for conservation, as well as forestry plantations based on *Eucalyptus* and *Corymbia* spp. In contrast to other regions where there are limited numbers of Myrtaceae plant hosts to manage, in Australia there is a multiplicity of species belonging to that family, and consequently more limited options with which to respond to this biological invasion. The large number of plant species that could be impacted, the putative large variation in susceptibility, both within and between species, and the significant differences between field and laboratory responses of pathosystems [14] make it extremely difficult to approach this problem with systematic foresight.

### Modelled risk

The CLIMEX model presented in this paper, which combined updated distribution as well as biological data for *P. psidii* s.l. gleaned from our experimentation, extends the estimated global area at risk from this invasive fungus into cooler climates than some previously published modelling attempts [6], [18], [19]. The global climatically suitable envelope for *P. psidii* s.l., based on the modelled EI values, spans the wet tropical and subtropical regions of the world with moderate temperatures, and includes some cooler regions with a mild Mediterranean climate. In contrast, the GI_A_ for *P. psidii* s.l., which only takes into account the potential for growth in favourable conditions and not the potential survival during stressful periods, indicates that there is an opportunity for seasonal development of the rust to occur over a much larger area of the world than that indicated by positive EI values. These additional areas identified by the GI_A_, however, are unlikely to be suitable under current climates for persistent rust populations to become established. Accordingly, the potential impacts in these climatically marginally suitable areas are likely to be limited. Where the requirement for a minimum annual heat sum will not be met, it is likely that in these areas any infection will not develop to sporulation unless the host plants are located in a warm microsite.

### Model reliability

The potential distribution model for *P. psidii* s.l. accords with all known locations where field infections have been noted. The location records in Japan, China and Australia were collected after the model stresses had been fitted [49], thus providing strong geographically independent support for the model. Given the rate at which biological invasions tend to proceed, it is unsurprising that there are large areas that are projected to be climatically suitable for establishment by *P. psidii* s.l., but for which there are no distribution records. For example, there are large areas in central Africa and the Mediterranean that appear climatically suitable for *P. psidii* s.l., and where at least some suitable myrtaceous hosts are present [14], but for which there are no records of *P. psidii* s.l. These areas are climatically similar to areas occupied by *P. psidii* s.l., as indicated by the Köppen-Geiger zonations [43]. There are numerous possible explanations for the lack of records in these apparently climatically suitable areas, including a lack of introduced propagules, environmental and demographic stochasticity, poorly-suited hosts, and a lack of appropriate search effort.

The significant uncertainty surrounding the wet and hot-wet tolerance limits for *P. psidii* s.l. should be kept in mind when considering the modelled risks in the tropics. Should this aspect of the model become critical to some important decisions, then transect surveys using susceptible trap plants could be undertaken along critical climatic gradients [56]. The modelled poleward limits of *P. psidii* s.l. should be considered indicative, rather than prescriptive. For example, where *P. psidii* s.l. climatic suitability appears to diminish in the Australian Alps and in Tasmania, the climatic gradients are extremely steep in relation to the scale of the climate data used in this exercise, and due to the precision of the climatic dataset [63] there may be favourable microhabitats within the areas modelled as unsuitable.

### Comparison with previous modelling efforts

The modification of the original Kriticos and Leriche model [49] to encompass empirical results on the germination of urediniospores of an Australian accession of *P. psidii* s.l. under different temperatures had a trivial impact on the extent of the modelled potential geographical range. Including the new experimental data in the model improved the apparent relative climatic favourability of warm temperate climates *within* the modelled potential geographical range.

Compared to the models of Booth et al. [18] and Booth and Jovanovic [19], the potential distribution model presented here draws on a broader range of distribution data, and benefits from the inclusion of information drawn from experimental observations of the germination and growth of the fungus [33], [34]. Our model indicates a significantly greater ability of the organism to tolerate cold stress than these other models.

The model of Magarey et al. [20] is broadly analogous to the GI_A_ in CLIMEX, and differs from the EI in ignoring those climatic stress factors that may reduce the potential for long-term persistence at a location. The net result of our study is a model that displays a generally more conservative risk picture than that portrayed by Magarey et al. [20] in regions experiencing seasonally hot dry conditions and continental climates, e.g., north-eastern USA and northern China. Curiously, the model of Magarey et al. [20] indicates that south-eastern Australia (south of Sydney) is unsuitable for *P. psidii* s.l. and yet more extreme continental climates (north-eastern USA and northern China) are suitable.

Elith et al. [21] presents results of several MaxEnt models, exploring the taxonomic uncertainty within the *P. psidii* s.l. complex. The “Puccinia_94” results in Fig. 2 of Elith et al. [21], were generated using data for a grouping loosely referred to as *P. psidii* s.l. and are taxonomically the most comparable to the CLIMEX model presented here, and generally accord reasonably well with our results. However, there are some notable contrasts in the MaxEnt and CLIMEX models. The CLIMEX results for current climate indicate that southern Tasmania and the South Island of New Zealand are unsuitable for establishment, and indicate a larger area at risk in WA than indicated by the MaxEnt Puccina_94 model [21]. The latter indicates that warm moist tropical environments such as Irian Jaya are climatically unsuitable, which appears at odds with experimental data [33], [34]. Illogically, the two Uredo models in Elith et al. [21], which, by definition are based on a smaller environmental envelope than Puccinia_94, result in a significantly larger area modelled as suitable in Australia. By changing the background with the input locations, Elith et al. [21] may have confounded modelling artefacts with the taxonomic treatment effects in their results. The resulting MaxEnt Uredo maps indicating that xeric regions of Australia may be marginally suitable for invasion and that Alpine regions are highly suitable for invasion are clearly nonsensical. The reason for these results, which we consider implausible, clearly requires further investigation.

### Species richness hypothesis

The map of species richness of Myrtaceae within the suitable climatic envelop for *P. psidii* s.l. in Australia presented in this paper shows the areas where epidemics may be more likely to be frequent and severe due to large numbers of different Myrtaceae species present. We hypothesise that regions rich in Myrtaceaeous species are more likely to contain one or more hosts susceptible to *P. psidii* s.l. Complementary development rates (e.g., [64]) and phenologies [65] amongst these hosts would also provide *P. psidii* s.l. with suitable foliage for infection throughout several periods of the year. All else being equal, higher rust inoculum loads might develop in these areas, and thus pose a greater threat to susceptible hosts than in areas of lower Myrtaceae richness. By comparing the climate suitability map for *P. psidii* s.l. with the map of Myrtaceae species richness, it is possible to identify hypothetical hotspots for epidemics where the EI is projected to be optimal and the highest diversity of Myrtaceae is found. Those hotspots are located in a narrow strip along the eastern coast of NSW, including the Sydney Basin, in the Brisbane and Cairns areas in Queensland and in the coastal region from the south of Bunbury to Esperance in WA. Sites within these areas would be ideal to implement long-term monitoring experiments to quantify the impacts of *P. psidii* s.l. [66] to guide future management responses.

### Additional threats to vulnerable species

*Puccinia psidii* s.l. poses a new threat to plant species of the family Myrtaceae that are already recognised as threatened by a range of other factors under the Federal EPBC Act and/or various state Acts in Australia [53]. The map of the threatened Myrtaceae species in combination with the climatic suitability and Myrtaceae species richness maps presented in this paper could facilitate the identification of areas where threatened species are most at risk from *P. psidii* s.l., and thus deserving surveillance and management attention. It remains to be established whether *P. psidii* s.l. poses an extinction risk to already threatened species, or whether background levels of resistance are sufficient for plant communities to adapt to the presence of this invasive rust.

### Threats to forest production

*Puccinia psidii* s.l. has so far only been reported on a few *Eucalyptus* spp. in native forests in Australia [67], [68], and has been found only once severely affecting seedlings in a eucalypt plantation (A. Carnegie, personal communication). Nonetheless, there is potential for disease development in forestry plantations in Australia because nearly 80% are located within the climatic suitability envelop for *P. psidii* s.l. Under current climate, the most northerly portion of Tasmania, an area dense with eucalypt plantations, appears suitable for *P. psidii* s.l. The only plantations that have no apparent risk of supporting persistent rust populations are those located in the Northern Territory.

### Spread in Australia

Considering the rapid spread of *P. psidii* s.l. via the nursery industry and wind-borne spores since its introduction in Australia [12], [69], it would seem inevitable that the risk areas identified by our CLIMEX model will eventually become invaded. At present, the spread of the fungus has been limited to eastern Australia, which is isolated by deserts from the areas of WA that contain significant Myrtaceae diversity and eucalypt plantations. These deserts and the prevailing western wind patterns may slow the natural spread of *P. psidii* s.l. into Western Australia, supporting the efforts of biosecurity managers there to prevent its spread via human movement of infected plant material. At present, only a small area of Tasmania appears climatically suited for persistent populations of *P. psidii* s.l., although most of Tasmania is suitable to support some population growth. Under a warming climate we would expect this area of suitability to increase. The separation of Tasmania from mainland Australia via the Bass Straight may provide a hindrance to the spread of *P. psidii* s.l. into Tasmania, though the presence of islands in Bass Strait may provide the opportunity for a stepping-stone invasion pathway. Tasmanian biosecurity managers may wish to conduct routine surveillance of these islands with a view to eradicating isolated infections as a means of slowing the spread of *P. psidii* s.l. into Tasmania.

### General applicability of the methods

The modelling and analytical methods demonstrated in this paper can help biosecurity and conservation managers to identify areas at heightened risk from IAS. These methods add to pest risk mapping methods available to assist these managers to gauge the size or value of the threats and to target interventions to manage them [1], [2], [70]. The pre-border pest risks from *P. psidii* s.l. to Africa, most of Asia, south-western Europe and New Zealand were identified using well-established niche modelling methods. The post-border example for Australia presented in this paper demonstrates how these broad scale bioclimatic risk patterns can be downscaled to identify and quantify relative risks to assets within the climatically suitable range. By combining bioclimatic niche modelling with non-climatic factors such as host species richness, we are able to move beyond simply identifying invasion risks, to provide some insight into the potential impacts that invasive species might have if they expand their range into climatically suitable areas, and distinguishing between invasiveness and impact [71]. In so doing we are able to inform biosecurity policies regarding the management of spread of IAS, and protection of valued assets.
